# Protein Therapeutic Quality Control Using Multi-Attribute Method (MAM): Challenges and Current Practice in cGMP Environments

**DOI:** 10.3390/ph19091444

**Published:** 2026-09-11

**Authors:** Zhiqi Hao, Christopher Yu, Anja Bathke, Jack Yim, Alexander Buettner, Feng Yang, Dietmar Reusch, Yi Yang

**Affiliations:** 1 Pharma Technical Development, Genentech, A Member of the Roche Group, South San Francisco, CA 94080, USA; xiaolany@gene.com (C.Y.); yim.jack@gene.com (J.Y.); yang.feng@gene.com (F.Y.); 2 Pharma Technical Development, F. Hoffmann-La Roche Ltd., 4070 Basel, Switzerland; anja.bathke@roche.com; 3 Pharma Technical Development Analytics, Roche Diagnostics GmbH, 82377 Penzberg, Germany; alexander.buettner@roche.com (A.B.); dietmar.reusch@roche.com (D.R.)

**Keywords:** multi-attribute method (MAM), targeted attribute quantitation (TAQ), new peak detection (NPD), protein therapeutic quality control

## Abstract

The increasing complexity of modern protein therapeutics and the demand for faster development of new therapeutics require not only advanced analytical tools that provide in-depth understanding of the product quality attributes (PQAs) that are crucial for safety and efficacy but also the implementation of a control strategy to ensure that the released drug product meets the desired quality profile. The multi-attribute method (MAM), a liquid chromatography–mass spectrometry (LC-MS) approach, has emerged as a transformative solution that provides site-specific monitoring of multiple critical quality attributes (CQAs) in a single workflow, replacing several conventional profile-based assays. The MAM achieves comprehensive quality oversight through two primary mechanisms: targeted attribute quantitation (TAQ) for the simultaneous quantification of predefined known modifications, and new peak detection (NPD) for identifying unforeseen impurities, thereby establishing a robust, “double-layered” control strategy. This article reviews the practical challenges and current industry practices for successfully implementing the MAM within current good manufacturing practice (cGMP) environments. The successful transition of the MAM from a specialized characterization tool to a validated quality control (QC) cornerstone relies on a framework built upon four foundational pillars: stringent instrument qualification paired with compliance-ready informatics, proactive and strictly scheduled instrument maintenance, rigorous system suitability testing (SST), and phase-appropriate method validation and transfer. The challenges of global method transfer and the solutions to mitigate inter-laboratory variability are discussed. Finally, the review explores the emerging trends shaping the future of the MAM in QC and the critical role of the MAM in facilitating real-time release testing (RTRT) in next-generation autonomous manufacturing.

## 1. Introduction

The therapeutic landscape for protein-based medicines has seen an extraordinary evolution over the last decade, with monoclonal antibodies (mAbs), fusion proteins, and bispecific constructs becoming the cornerstone of modern treatment for oncological and autoimmune diseases. As these modalities grow in structural complexity, the analytical burden of ensuring their safety, efficacy, and consistency has increased commensurately. At the same time, there is increasing demand for faster development of new therapeutics and more efficient, cost-effective manufacturing processes. The quality by design (QbD) approach recommended by regulatory agencies requires not only an in-depth understanding of the product quality attributes (PQAs) that are crucial for safety and efficacy but also implementation of a control strategy to ensure that the released drug product meets the desired quality profile [1,2,3,4]. Conventional quality control (QC) strategies for biopharmaceuticals typically rely on a suite of “profile-based” methods, such as ion exchange chromatography, capillary electrophoresis, reversed-phase chromatography, and imaged capillary isoelectric focusing. While these techniques are robust, they use either UV or fluorescence detection and are unable to identify the specific cause of the change in profile-based quality attributes. In addition, significant resources are required to maintain multiple instruments and distinct validated methods [5,6]. To address these limitations, the multi-attribute method (MAM) has emerged as a transformative liquid chromatography–mass spectrometry (LC-MS) approach. The MAM leverages the high resolution and mass accuracy of modern mass spectrometers to provide a granular, residue-level view of a molecule’s critical quality attributes (CQAs) in a single workflow. By transitioning from the monitoring of broad chromatographic profiles to the direct quantification of specific amino acid modifications, the MAM offers an unparalleled depth of product understanding, aligning seamlessly with the industry’s push toward modernized manufacturing and stringent regulatory standards [7,8,9,10,11,12,13,14,15].

**Figure 1:**
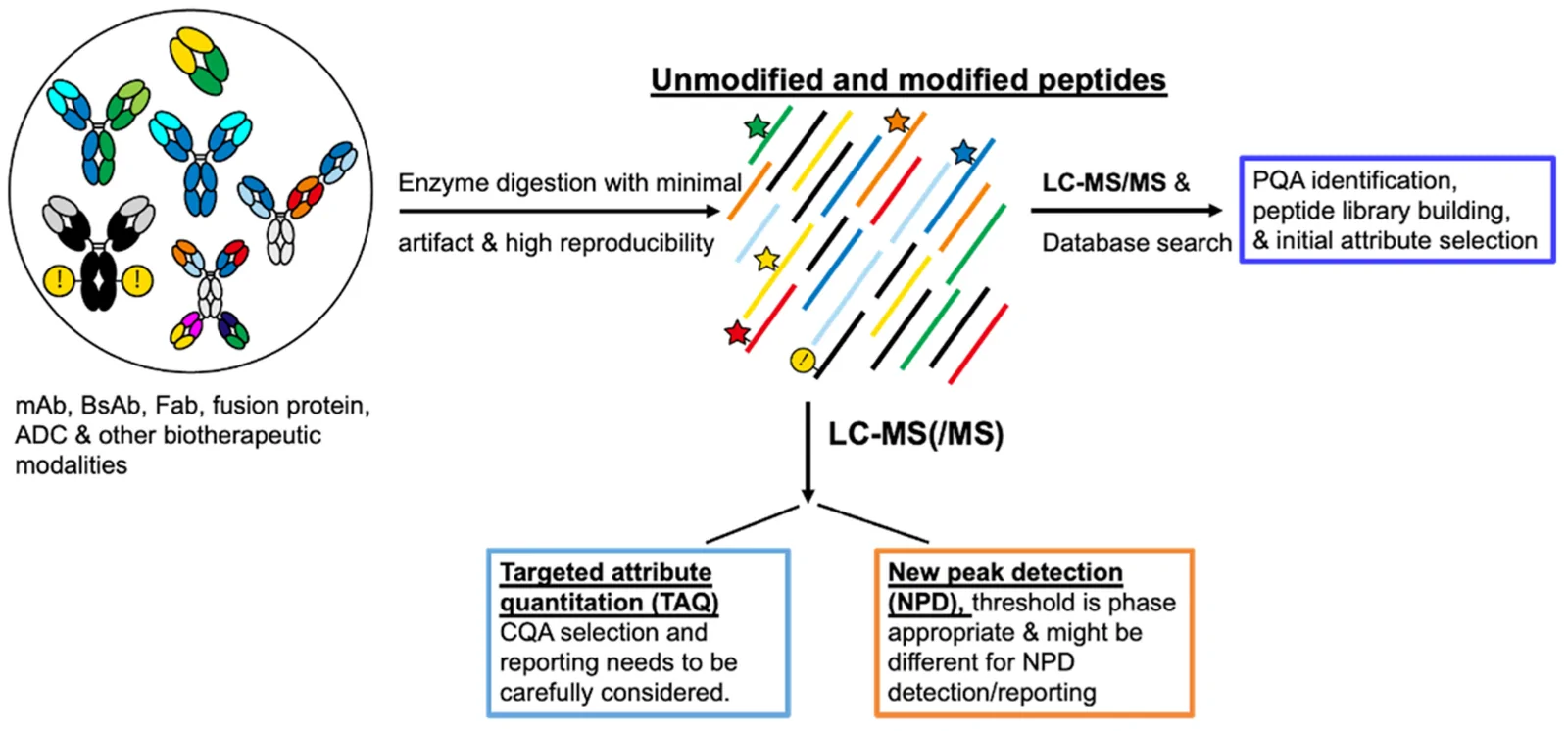
Generic MAM workflow for biotherapeutic modalities that enables targeted attribute quantitation and new peak detection. Adopted from Yang et, al. 2023 [13] with permission.

The MAM is a peptide-mapping-based approach that utilizes high-resolution accurate-mass (HRAM) data to achieve two primary objectives: targeted attribute quantitation (TAQ) and new peak detection (NPD) [13,16,17]. Targeted attribute quantitation (TAQ) is the monitoring arm of the workflow. It focuses on the simultaneous quantification of a predefined set of known CQAs, such as oxidation, deamidation, glycation, and N-terminal cyclization. A peptide library or “workbook” is established that contains the precise retention times, mass-to-charge ratios (*m*/*z*), and fragmentation patterns of both modified and unmodified peptides. TAQ enables the automated calculation of the percentage of modification for each attribute by comparing the peak area of the modified peptide to its unmodified counterpart. This site-specific monitoring provides a level of detail that traditional methods—which might only show a general increase in “acidic variants”—cannot match [13,17,18,19]. Complementing TAQ is new peak detection (NPD), which serves as a comprehensive purity test. While TAQ monitors what is expected, NPD is designed to catch the unexpected. By performing an automated, unbiased comparison of the LC-MS chromatogram of a sample against a designated reference standard, NPD can flag any new or significantly altered peaks that fall outside of pre-established thresholds. This function is vital for detecting unforeseen process-related impurities, sequence variants, or degradants that might be invisible to TAQ and conventional UV-based assays [13,17,20,21]. Together, TAQ and NPD provide a “double-layered” control strategy that ensures both the consistency of known attributes and the absence of unexpected impurities and product variants (Figure 1).

The ability of the MAM to monitor multiple, different types of CQAs in a single LC-MS injection, and therefore replace multiple traditional assays, represents a significant paradigm shift. In a typical QC laboratory, maintaining three to five different types of instruments for various purity and identity tests is costly and labor-intensive. The MAM streamlines this by reducing the number of standard operating procedures (SOPs), minimizing sample preparation time through automation, accelerating the data review process via integrated informatics, and reducing resources (personnel and time) for instrument lifecycle management. This consolidation not only reduces the cost of goods but also eventually shortens the “bench-to-market” timeline, which is especially critical for the rapid release of breakthrough therapies. Beyond efficiency, the MAM is a physical realization of the QbD framework as outlined in ICH Q8, Q9, and Q10. QbD emphasizes that quality should be built into the product through a deep understanding of the manufacturing process and its impact on CQAs. Because the MAM provides direct, site-specific data, it allows process scientists to correlate specific manufacturing parameters (e.g., pH, temperature, or media components) with individual molecular modifications. This enables the establishment of scientifically justified control strategies and facilitates “real-time” process monitoring, moving the industry away from a “testing-to-document” mindset toward a proactive “testing-to-control” approach.

Since its introduction a decade ago, the trajectory of the MAM in the biopharmaceutical industry has moved from being a specialized tool for characterization to a validated cornerstone of late-stage development and commercial manufacturing [12,13]. The initial validation and deployment of the MAM were led by a small group of early adopters who demonstrated that LC-MS could meet the rigorous standards of current good manufacturing practice (cGMP). These pioneers proved that with the right controls—such as system suitability testing and automated data processing—the MAM could achieve the precision and accuracy required for batch release [17,22]. A major indicator for a robust QC method is its transferability across global sites. Recent multi-laboratory studies have shown that the MAM is remarkably robust, with high correlation of results across different MS platforms and geographic locations [23]. This transferability is essential for global pharmaceutical companies that manufacture products in multiple regions and must ensure that a batch released in Europe is identical to one released in Asia or North America.

The transition of the MAM into the QC space has been characterized by active dialog with health authorities. The FDA’s Emerging Technology Program (ETP) has played a pivotal role in reviewing MAM submissions, focusing on the validation of NPD algorithms and the bridging of data between conventional methods and MS-based assays. Furthermore, the USP officially introduced a General Chapter <1060> titled “Mass Spectrometry-Based Multi-Attribute Method for Therapeutic Proteins.”, providing guidance for assay validation and method transfer. In addition to the peptide mapping MAM approach, intact and subunit-level LC-MS analysis and use of the MAM in process analytical technology (PAT) are also addressed [24].

This review focuses on the practical challenges and current industry practices for successfully implementing the MAM within cGMP environments. It is an industry perspective and implementation review rather than a conventional literature-based review. Our own established workflows—such as localized and enterprise informatics architectures, system suitability protocols, and phase-appropriate validation approaches—are included as specific case studies to evaluate and discuss the real-world deployment of MAM into routine QC operations.

## 2. Implementing MAM in a GMP Environment

The implementation of the MAM in a GMP environment builds upon a robust framework that extends beyond standard analytical performance, focusing on four pillars: a validated informatics infrastructure, proactive maintenance strategies, stringent system suitability testing (SST), and a phase-appropriate validation approach.

### 2.1. Instrument Qualification and Informatics

Transitioning MS from a research environment to a regulated QC laboratory requires a stringent instrument qualification framework combined with a validated informatics system to ensure the highest level of data integrity. Instrument qualification is the documented process of proving that an analytical system is fit for its intended use. For the MAM, this follows the classic four-stage lifecycle as defined by USP Chapter <1058> [25]:

- Design Qualification (DQ): The initial step where the lab defines the functional and operational specifications of the LC-MS system. These specifications define what the hardware (such as an LC pump, autosampler, and a mass spectrometer) must be able to do to perform the required analysis. They also define how the instrument integrates into the laboratory environment and facility and meets regulatory or business standards on software and data integrity.
- Installation Qualification (IQ): This phase confirms that the system is delivered as specified and installed correctly in a suitable environment. Key elements include verifying power, gas supplies (high-purity Nitrogen), and documenting firmware versions to ensure they match the validated state.
- Operational Qualification (OQ): The system must demonstrate it can operate within the manufacturer’s specified limits. For the MAM, OQ involves testing core parameters like mass accuracy, detector linearity, and retention time reproducibility. It also includes “failure mode” testing to verify how the system handles communication errors or power interruptions.
- Performance Qualification (PQ): PQ verifies the system’s ability to perform the specific MAM assay consistently under routine conditions. This usually involves a “System Suitability Test” (SST) using a representative protein digest to ensure the entire workflow meets predefined acceptance criteria for TAQ and NPD.

The MAM generates massive datasets and relies on sophisticated informatics that must be “compliance-ready” under 21 CFR Part 11 and EU GMP Annex 11. Within a multi-site network, Roche employs enterprise-grade solutions to ensure robust compliance with these regulatory frameworks. These software solutions are designed to incorporate critical technical controls, providing a reliable foundation for managing data integrity and regulatory adherence across complex workflows:

- Audit Trails: Automated, time-stamped records of “Who, What, When, and Why” for every data modification or method change.
- Electronic Signatures: Unique identifiers irrevocably linked to records, often following a “Submit, Review, Approve” workflow.
- Role-Based Access Control (RBAC): Restricting system access to authorized individuals and ensuring “Separation of Duties” (e.g., an analyst cannot also be the reviewer).
- Data Integrity (Checksums): Use of cryptographic hashes to ensure that raw MS data files have not been tampered with or corrupted during transfer.
- Enterprise Data Management: Dedicated “Production” (PROD) and “Development” (DEV) data vaults are utilized, where methods are locked upon transfer to the QC environment to prevent unauthorized modifications.

The following platforms are currently available software that fully meet 21 CFR Part 11 and Annex 11 requirements for MAM operations (Table 1).

Two distinct informatics architectures are used at Roche to support the integration of the MAM into QC environments: a localized small network and a centralized enterprise infrastructure. Both configurations are specifically designed to ensure rigorous data integrity, system security, and regulatory compliance.

The initial deployment utilizes a localized Chromeleon small network connecting two GMP-qualified instruments, two non-GMP instruments, and multiple remote workstations. This architecture operates with the host computer of a GMP instrument acting as the central hub. To enforce security, Microsoft Windows Active Directory Group Policy Objects (GPOs) are applied directly to the GMP host computers. These GPOs restrict system access to designated user groups, prohibit the use of removable storage devices, and remove standard desktop icons—such as the Recycle Bin, My Computer, and Network—to prevent unauthorized system actions. All raw data and metadata are maintained exclusively as local electronic records, eliminating the need for physical printouts. A secure, validated IT backup solution systematically duplicates all data and audit trails, ensuring backup files remain identical to the originals. Rather than employing periodic data relocation, all information is archived locally for full lifecycle accessibility, supported by continuous automated backups of data and system images. Finally, method design and validation activities are strictly segregated using a controlled folder hierarchy.

Subsequent implementations at other Roche sites transitioned to a centralized enterprise CDS solution. Each LC-MS system utilizes a dedicated control PC integrated into the secure corporate network. This framework enables centralized data storage on dedicated servers, facilitating multi-location data evaluation via terminal servers. Domain controllers manage data flow, supplemented by a robust backup strategy consisting of weekly full backups and daily differential backups to secure network drives. This architecture strictly segregates a testing and development environment for method design from a production environment dedicated exclusively to GMP analytics.

Within GMP operations, data management necessitates a rigid, sequence-based workflow. Sequences require standardized nomenclature and placement in predefined folder structures prior to any data acquisition. An automated “Ready Check” verifies sequence plausibility and system resources before initiating the analytical queue. Analytical methods—comprising instrument and data processing methods as well as report templates—are established in the development environment and formally migrated to the production environment by designated key users. Following transfer, methods are strictly version-controlled to preserve their validated state. Substantive technical modifications are re-evaluated within the development environment prior to re-transfer, whereas minor or editorial adjustments can be executed directly within the production environment by creating a new method version. In all cases, any method update requires a documented reason for change, key user authorization, and new electronic signatures. Furthermore, all sequence data, including metadata and interrupted or aborted runs, are automatically saved into administrator-configured folders upon the completion of each injection.

User access is governed by a role-based access control (RBAC) model administered via the Chromeleon User Manager. Personnel receive specific logon roles (e.g., routine GMP users, development analysts, or key users) mapped to corresponding functional privileges. Laboratory reviewers are restricted from moving or renaming sequences. Neither regular GMP users nor application administrators possess the technical privilege to delete GxP raw data or metadata from the system. Data deletion capabilities are strictly restricted to backend IT system administrators under formal global IT change management processes. While pre-approval sequence modifications are permitted, they are recorded in the audit trail. When a GMP user “Submits” a sequence, it is locked. However, if corrections or manual re-integrations are required according to test procedures, the reviewer has the specific system privilege to execute a “Remove Submit”. This lifts the data lock and allows the GMP user to re-process the data. Following data review, sequences are mandatorily locked into a “read-only” status; any subsequent alterations require a formal event management process involving the process owner and laboratory informatics personnel. Ultimately, all functional activities, including manual integration and method transfers, are captured in detailed audit trails, which serve as a mandatory component of the secondary review process.

Beyond specific vendor platforms, implementing the MAM industry-wide requires a unified framework for data integrity, regulatory compliance, and software interoperability [26]. Compliance relies on strict adherence to the ALCOA principles. These are outlined in FDA Data Integrity Guidance, 21 CFR Part 11, EudraLex Volume 4 Annex 11 guidelines for computerized systems, as well as the newly introduced USP General Chapter <1060>, which provides dedicated guidelines for MAM software compliance, method qualification, validation, and analytical transfer. From an informatics standpoint, eliminating manual data transfers and securing an end-to-end digital thread from MS acquisition to reporting is critical; in fact, USP <1060> explicitly emphasizes that if data acquisition and reporting are performed in separate software packages, additional mechanisms must exist to maintain and track data integrity between them. Interoperability should enable seamless integration of complex MS datasets into enterprise laboratory information management (LIMS) and electronic laboratory notebook (ELN) systems without compromising the audit trails or raw data access mandated by 21 CFR Part 11 and Annex 11. Furthermore, method transfer poses data management risks if software ecosystems or IT architectures are altered across sites, departments, or product phases, a challenge frequently addressed in implementation reviews [26]. Switching informatics platforms disrupts the digital thread and the analytical control strategy. Maintaining a consistent data architecture throughout the lifecycle is vital, as fragmented systems hinder the ability to re-process historical full-scan data seamlessly. This retrospective capability—evaluating historical files for newly identified attributes without generating new lab data—aligns directly with the product lifecycle management and analytical procedure development principles established in the ICH Q14 and ICH Q2(R2) guidelines.

### 2.2. Instrument Maintenance

Implementing the MAM in a GMP QC environment necessitates a shift from reactive instrument maintenance to a rigorously scheduled, proactive preventative maintenance (PM) program. Mass spectrometers are highly complex compared to standard liquid chromatography (LC) or UV detectors; therefore, preserving the sensitivity, isotopic resolution, and mass accuracy needed to detect low-abundance peptide modifications requires strictly defined maintenance intervals.

In regulated QC settings, maintenance is categorized into routine operational checks and periodic comprehensive preventive maintenance and operational qualification (PMOQ). Routine maintenance, performed daily or weekly based on instrument types, focuses on mass calibration and the electrospray ionization (ESI) source. Because non-volatile salts and residual detergents frequently accumulate in the ESI source, regular cleaning of the spray shield and capillary entrance is essential to prevent sensitivity loss and signal drift. Additionally, MAM performance for TAQ and NPD demands a mass error strictly within 10–20 ppm. Automated mass calibration, alongside functional checks using reference materials, ensures stable system performance. Instruments failing predefined criteria, such as inadequate peptide resolution or insufficient signal-to-noise ratios, must be flagged for maintenance prior to generating GMP data.

Comprehensive PMOQ, performed regularly by a certified service engineer, addresses both MS and LC components. Because NPD algorithms require strict retention time windows to differentiate known attributes from potential impurities, LC stability is paramount and necessitates replacing high-wear parts like pump seals, needle seats, and injection valves. MS maintenance encompasses verifying system audit trails, annually servicing roughing and turbomolecular pumps, and monitoring detector operating voltages to identify fatigue and preserve the linear dynamic range necessary for CQA quantitation. Operational qualification (OQ), the same tests performed as part of the instrument initial operation qualification, is performed to ensure the instrument is operating as intended and within specification before returning for GMP use.

Despite proactive PM, sudden issues like vacuum leaks, detector fatigue, or ion source contamination can still occur. Given the high stakes of QC batch releases and the complex, time-consuming nature of MS troubleshooting, relying on a single instrument presents a significant business risk. Facilities can mitigate this by maintaining an identical, fully qualified reserve system to prevent delays. Furthermore, following any troubleshooting, significant repair, or software patch, a complete functional check is mandatory to verify that the system has successfully returned to its validated state. We have developed an in-depth evaluation using an in-house protein digest standard with a set of criteria for optimum instrument performance. This approach has been used both in the development lab and in the QC lab to evaluate instrument performance.

### 2.3. System Suitability Test

System suitability testing (SST) used to assess MAM suitability is more complex than for standard HPLC. It must verify the performance of three distinct components: the sample preparation (enzymatic digestion), the LC separation, and the MS detection and quantitation, for the method’s two primary functions: TAQ and NPD.

There are several options for assay control material used to perform SST. Commercially available synthetic peptide mixture or pre-digested protein standards can be used to assess overall LC-MS performance, although they do not provide information on sample preparation performance, including protein denaturation, reduction and alkylation of cysteines, and trypsin digestion.

A well-characterized batch of the actual therapeutic protein (e.g., NISTmAb or an internal reference standard) that undergoes the same enzymatic digestion as the samples can be used as the benchmark for “Expected” peaks during TAQ and the baseline for “New” peaks during NPD [27,28]. SST based on the analysis of a product-specific reference standard is highly recommended by the industry leaders [29]. In the MAM consortium interlaboratory study using NIST mAb, the results show that the behavior of one peptide (e.g., an arbitrary Reference Peptide) in the MS source may not be predictive of the behavior of a different peptide (e.g., the attribute peptides). For this reason, incorporation of attributes of interest into the system suitability test when performing the MAM with TAQ is recommended [30]. Assay control material used for the NPD workflow can include a negative and a positive control. In a recently published NPD workflow, comparison of bracketing injections of the product-specific reference standard serves as negative control, whereas the comparison of the reference standard with the same reference standard spiked with the Pierce™ Retention Time Calibration (PRTC) standard peptides at 0.5 pmol spiking level serves as positive control [20].

It is also feasible to use an in-house, well-characterized antibody as an assay control for overall method performance verification. This assay control is prepared alongside samples of interest in each analysis to demonstrate performance of both sample preparation and LC-MS analysis. In addition to this universal assay control, one can also include a product-specific reference standard in each assay. This provides the opportunity for the control of specific quality attributes that are unique to the product molecule and may not be adequately controlled by the universal SST [22]. In addition, the product-specific reference standard is also used as a negative control for NPD SST. For one of our products, the positive control used for NPD is the same molecule that contains a sequence variant [21]. A potential positive control for NPD could also be heavy-labeled peptides spiked into the same product-specific reference standard.

The SST parameters for the MAM must demonstrate that the system can accurately quantify known attributes while maintaining the sensitivity and selectivity to detect unknown impurities. The parameters for TAQ should include mass accuracy and signal intensity in addition to the parameters to monitor the performance of chromatography, such as retention time reproducibility. SST parameters for TAQ and overall method performance of our platform method include mass accuracy, signal intensity, and relative quantitation of low-abundance peptides at pre-defined retention time windows, which allows us to assess both MS and LC performances [21,22]. The parameters for NPD thresholds should be empirically determined individually for specific processes and products as well as for different instruments and software platforms [27,28]. Reported parameters using “Spiked Controls” are detection threshold (a pre-defined intensity threshold, e.g., 0.1% to 1.0% of the base peak chromatogram), fold-change sensitivity (the system must demonstrate the ability to detect a “new” peak at a specific fold-change, typically > 5-fold or 10-fold increase relative to the reference), and the absence of “New Peaks” in a comparison of the reference standard against itself, ensuring the software does not report noise as a product variant [20,21]. The system would pass suitability testing if all peptides (spiked into product-specific reference standard) were appropriately detected as new or changed peaks and no unexpected new peaks arose. Such a system suitability control would provide a consistent NPD performance metric to be incorporated into every MAM sequence, thereby providing a means to actively monitor NPD performance [27,28]. Table 2 lists the comprehensive SST parameters of our platform MAM.

### 2.4. Method Validation

To implement the MAM in a regulated environment, method validation is essential to ensure the method is suitable for its intended purpose [15,17,22]. The validation of the MAM follows a phase-appropriate approach, aligning with ICH Q2(R2) guidelines, but with specific adaptations for MS-based technology.

A typical MAM test procedure involves two essential components: TAQ and NPD. TAQ focuses on monitoring specific, predefined PQAs such as deamidation, oxidation, glycosylation, and N/C-terminal variants. Following enzymatic (typically trypsin) digestion, samples are separated by LC and detected by MS. Specific peptides—identified during initial characterization—are quantitatively monitored using extracted ion chromatograms (XICs). The method calculates the abundance of modified peptides relative to their unmodified counterparts to determine the percentage of modification (e.g., % Oxidation or % Deamidation). High-resolution mass analyzers are essential to distinguish the target peptide from co-eluting species based on exact mass. To ensure consistent peak picking across thousands of injections, the method utilizes retention time windows or alignment algorithms. The mass detector must maintain a wide linear range to quantify low-abundance impurities alongside dominant native peptides. Validation for TAQ mirrors traditional purity assays, covering parameters such as specificity, accuracy, precision, response, range, and quantitation limit for the TAQ component [31].

NPD serves as an untargeted purity test, acting as a “digital comparison” to ensure no unexpected species or changes are present in the sample. It is the primary tool for detecting process drifts or contaminants not covered by TAQ. NPD uses automated software to compare a sample’s MS1 profile against a reference injection to identify any new or significantly changed peaks that fall outside a defined threshold of intensity or mass-shift. The method must compensate for minor chromatographic shifts to prevent false-positive “new peaks” caused by misaligned signals. Sophisticated algorithms are required to differentiate true chemical signals from electronic noise or baseline fluctuations. Validation of NPD is arguably the most complex hurdle for the MAM in a GMP environment. While traditional “attribute monitoring” is a targeted approach (looking for what you know), NPD is untargeted (looking for what you do not know), making it a “Limit Test” under ICH Q2 guidelines. NPD validation includes specificity (false positive/negative rates) and detection limit [31].

Due to the complexity of the method and the fact that multiple attributes are being measured, validating a MAM is significantly more complex than validating a traditional, alignment-based LC method. Despite these hurdles, progress has been made across various platforms. Ghosh et al. successfully executed a full validation of an LC-MS/MS method for antigen detection, adhering to current FDA and EMA regulatory frameworks [32]. Sokolowska et al. described the co-validation of a subunit LC–MS method for monitoring antibody oxidation for commercial product release and stability testing [33]. Hao et al. reported a detailed MAM validation on TAQ performance. The study described the MAM performance profile and addressed the major scientific challenges of MAM validation [22]. Xu et al. demonstrated the utility of a single-quadrupole QDa-based MAM for streamlining product characterization, process development, and routine QC [34]. A recent study details the co-validation of a UPLC-ToF MS-based MAM across three independent laboratories. The method was fully validated for identity testing and quantitative monitoring of two CQAs for a Peptide-Fc fusion protein drug QC release and stability testing [35].

Pohl et al. reported for the first time the creation and validation of a sophisticated NPD workflow by integrating automated tools to filter out instrument noise and chemical artifacts. The workflow can identify low-abundance impurities without reporting false positives [20]. Classified as a limit test under the revised ICH Q2(R2) framework, the validation of the untargeted NPD function primarily focuses on demonstrating adequate specificity and establishing a reliable detection limit (DL) [29,31].

To formally validate NPD, the industry standard relies on spiking product digests with standard mixtures of non-product synthetic peptides (e.g., the 15-peptide Pierce™ Retention Time Calibration mix) across multiple concentration levels, or using well-characterized sequence variant models and stressed samples [20,21,36]. Acceptance criteria for specificity require zero false-positive flags when comparing unspiked reference standards against themselves, alongside the systematic exclusion of routine method artifacts via a validated ‘Known Peak List’ (KPL) [21]. For the DL, 100% of the spiked control peptides or product variants at and above the defined reporting level must be consistently detected as ‘new’ or ‘changed’ peaks across replicate analytical runs [20].

The practical execution of NPD relies on the empirical optimization of three core software parameters to balance detection sensitivity with false-positive suppression:

- Intensity Threshold (IT): Defines the minimum baseline signal required for peak evaluation, typically set between 0.01% and 1.0% relative to the total ion chromatogram (TIC) or base peak chromatogram (BPC) [27,29].
- Fold-Change Detection (FCD) Threshold: Specifies the magnitude of intensity increase required to flag a peak as ‘changed’, typically established between 3-fold and 10-fold relative to the reference standard [27].
- Alignment Tolerances: To prevent false positives caused by minor instrumental drift, mass alignment tolerances are strictly set between 5 and 20 ppm, combined with retention time alignment windows of 0.2 to 0.8 min [20,37].

At Roche/Genentech, our platform MAM has been validated for the analysis of various attributes across multiple different products. The initial method validation was to evaluate the TAQ performance of the high-resolution LC-MS-based peptide map method for implementation in clinical QC. Using three products and 11 attributes of four CQA types, a comprehensive evaluation of performance characteristics such as specificity, response (linearity), accuracy, intermediate precision, repeatability, range, quantitation limit, and robustness was performed. The results demonstrated that the method effectively delivered the expected QC performance across multiple products and CQA types. Additionally, a robust system suitability was established for both TAQ and the overall performance of sample preparation and LC-MS. This validation provides a solid foundation for the method performance in clinical QC laboratories and for the lean validation approach applied later to the clinical phase products [22]. The first NPD validation was performed using a product-specific sequence variant model. The validation demonstrated that the NPD feature of the method provided suitable specificity and a good detection limit for detecting unexpected changes in the product with minimum false positives and established the system suitability criteria for NPD (in addition to the previously established system suitability for TAQ and overall method performance). Table 3 lists the performance characteristics and acceptance criteria for the validation of our platform MAM.

To date, the MAM has been validated at Roche/Genentech for multiple products spanning clinical development to commercialization. Historical qualification and validation efforts have been performed on representative Roche/Genentech pipeline molecules—including monoclonal antibodies (mAbs), bispecifics, dutafabs, and conjugates—providing a solid foundation for further applications. To accelerate the drug development process and streamline operations in early-stage clinical development, Roche / Genentech have adopted a lean validation approach, fully aligned with regulatory expectations outlined in ICH Q2(R2). This approach increases productivity, reduces workload, and enables faster timelines while ensuring compliance and validation for the intended purpose in early clinical phases, avoiding exhaustive validation activities required for later stages of development and commercialization. MAM lean validation leverages results of comprehensive qualification and historical qualification and validation data of molecules with similar characteristics as well, simplifying and accelerating method validation for early-stage clinical development. Specifically, once a method qualification has demonstrated that the MAM performance (including specificity, accuracy, response, precision, and robustness for TAQ, and specificity, detection limit, comparability, and robustness for NPD) meets the requirements for intended use, method validation can be limited to fewer performance characteristics. This approach allows flexibility for the integration of new product modalities or formats, where additional assessment of the lean validation strategy may be required to ensure applicability. The lean validation approach supports the efficient and compliant progression of drug development pipelines while adapting to evolving regulatory and scientific demands.

### 2.5. Method Transfer

As the industry transitions the MAM from characterization into QC and GMP environments, the successful transfer of these complex methods between different laboratories and geographic sites has become critical to ensure the readiness and long-term robustness of the method [29]. The primary hurdle in transferring the MAM between sites is the inherent complexity of the peptide mapping workflow, which is sensitive to a wider array of variables:

- Sample Preparation Variability: The “bottom-up” approach requires protein denaturation, reduction, alkylation, and enzymatic digestion. Manual execution of these steps is labor-intensive and highly prone to analyst-to-analyst variation, which can lead to artificial modifications (e.g., sample preparation-induced oxidation or deamidation) that confound the results [38,39,40].
- Instrumental Heterogeneity: The MAM results can be influenced by differences in mass spectrometer platforms (e.g., Orbitrap vs. Time-of-Flight) and even between identical models due to differences in source tuning, gas flows, and detector aging [13]. Maintaining consistent sensitivity for NPD is particularly challenging across different instruments [41]. Ensuring that automated software correctly integrates peaks across sites requires rigorous alignment of retention time windows and mass accuracy thresholds [42].

The industry has adopted standardized approaches to mitigate these challenges, focusing on automation, robustness, and bridging studies. To eliminate manual error, many labs are implementing robotic liquid handling systems for automated digestion. These systems ensure that reagents, temperatures, and incubation times are strictly controlled, significantly improving the reproducibility of TAQ across different sites [40]. Automated workflows using high-throughput buffer exchange tips have been shown to maintain superior performance and reduce hands-on time by over 80% [38,39,40]. In addition, standardizing informatics platforms, such as enterprise-wide implementation, provides a centralized hub for data management and helps maintain consistent peak integration and reporting across a global network.

Current industry practice emphasizes extensive analytical method validation prior to transfer. This involves evaluating all ICH Q2 performance characteristics, with a heavy focus on intermediate precision and robustness. Sites often use reference materials to benchmark performance and ensure that the transferring and receiving laboratories achieve comparable results on TAQ and NPD.

Song et al. reported the implementation of a new automated analytical platform for the multi-attribute method (MAM) across three different sites using Orbitrap instruments. This platform successfully integrated automated sample preparation and LC-MS-based MAM. The results demonstrated the consistency of the automated sample preparation and the LC-MS instruments across the different locations [38].

The complexity of high-resolution mass spectrometry (HRMS) often hinders its use in GMP-compliant commercial QC environments. To bridge this gap, Evans et al. developed a two-tiered approach: using HRMS for deep characterization during development, followed by a streamlined multi-attribute monitoring (MAM) method for routine QC utilizing the low-resolution MS system for identity (ID) testing, sequence variant control, and CQA quantitation. The ID-MAM was successfully validated across six laboratories (three analytical development labs and three QC labs) in four countries, proving to be robust and reproducible. Furthermore, the CQA results obtained with this method were comparable to the results obtained using high-resolution peptide mapping [43].

A recent study demonstrated reliability and reproducibility of the MAM across different high-resolution instrumental platforms. The study utilized three different mass spectrometers, two Orbitrap instruments and one TOF instrument, across two laboratories to quantify various product quality attributes. The datasets generated by the different mass spectrometers yielded consistent findings in measuring several key product attributes, including oxidation, pyro-Q formation, clipping, and glycopeptides. Furthermore, the study established correlations between the MAM and conventional analytical techniques, such as hydrophilic interaction chromatography–fluorescence detection (HILIC-FLD) and cation exchange chromatography–ultraviolet (CEX-UV) [23].

A formal method transfer for the MAM typically would follow a comparative testing or co-validation approach, as defined by ICH Q2(R2). The process begins with a comprehensive gap analysis and risk assessment phase. During this stage, teams must ensure instrument equivalence by verifying that the receiving site possesses qualified mass spectrometers, such as Orbitrap or Q-TOF platforms, with full IQ/OQ/PQ documentation. Additionally, because MS is inherently complex, extensive personnel training focused on the reproducibility of enzymatic digestion and specialized software workflows is necessary. The receiving site performs trial runs using the donor site’s specific protocols to identify potential site-specific variables, such as variations between different brands of trypsin. The transfer concludes with comparative testing. Both laboratories analyze the same homogeneous lots, including reference standards and stability samples. To confirm a successful transfer, the results must meet statistical equivalence within pre-defined acceptance criteria.

The inter-laboratory variability is minimized when donor and receiving sites utilize identical MS hardware platforms, standardized eWorkflows, and automated robotic liquid handling systems for digestion.

### 2.6. Additional Practical Challenges of MAM Implementation

While the multi-attribute method (MAM) offers transformative benefits for biopharmaceutical quality control, transitioning the technology into a routine cGMP environment requires addressing several practical considerations [26]. Importantly, industry experience demonstrates that these operational challenges are readily manageable when supported by a well-designed implementation framework [29].

- Instrument and Operational Costs: High-resolution mass spectrometry (HRMS) platforms require higher initial capital investment and maintenance overhead than standard optical detectors [44]. However, this expenditure is strategically offset by the long-term consolidation of multiple conventional assays (such as IEX, CE-SDS, and HILIC) into a single LC-MS workflow [17].
- Training Requirements and Skill Boundaries: Operating MS hardware has historically been viewed as highly complex. However, routine QC analysts do not need to be specialized mass spectrometrists [42]. By deploying fixed, locked data processing methods and establishing close collaborative networks between analytical development subject matter experts (SMEs) and QC teams, routine execution and data review become straightforward and highly reproducible [33].
- Data Analysis Complexity: The massive datasets generated by the MAM are efficiently managed through modern enterprise chromatography data systems (CDS) [13]. Automated reporting templates, pre-defined integration rules, and centralized client-server architectures eliminate manual data manipulation, ensuring strict 21 CFR Part 11 compliance and data integrity [29].
- NPD Management: Balancing sensitivity against the risk of false positives caused by sample preparation artifacts or baseline noise remains a challenge [27]. This can be effectively controlled by implementing comprehensive product-specific “Known Peak Lists” and automated system suitability gating to filter out routine method artifacts [21].

## 3. Trends and Future of MAM in QC

### 3.1. High-Resolution vs. Low-Resolution Instruments

Driven by the maturation of the MAM, mass spectrometry is expanding from characterization into routine QC. This transition necessitates a careful weighing of the advantages and disadvantages between high-resolution systems and lower-resolution single–quadrupole (SQ) detectors within a cGMP environment. Key factors influencing platform selection include:

- Mass Accuracy, Resolution and Target Specificity: HRMS provides superior resolving power essential for separating minimal mass shifts (e.g., deamidation at +0.984 Da) and trace species, achieving limits of quantitation (LOQ) as low as 0.002% compared to ~1% for lower-resolution SQ instruments in full-scan mode [44]. Crucially, USP General Chapter <1060> explicitly notes that lower mass resolution cannot resolve peptide isotopic peaks to establish charge state identity for multiply charged tryptic peptides (z > 1) [USP <1060>]. Consequently, lower-resolution mass spectrometry (LRMS) exhibits an absolute reliance on physical chromatographic baseline separation to maintain the specificity mandated by ICH Q2(R2) [26].
- NPD Capabilities: Untargeted (NPD) requires sufficient mass resolving power to accurately differentiate true product anomalies from baseline noise and sample preparation artifacts [24]. As established by the MAM consortium, the high mass accuracy (≤5 ppm) inherent to HRMS platforms is critical to confidently flag low-abundance unexpected peaks while suppressing false positives [27]. Because LRMS platforms operating under unit mass resolution lack the spectral purity required for untargeted screening, HRMS remains the preferred industry standard for cGMP-compliant purity testing [20,44].
- Operational Complexity, Infrastructure, and Cost: Compact LRMS detectors feature lower capital expenditure and function essentially as “smart optical detectors” [43]. Historically, HRMS systems were complex and required dedicated specialists. However, vendor innovations in modern benchtop HRMS platforms—featuring automated system suitability checks and simplified user interfaces—have lowered operational barriers for routine QC environments [15,29].
- Software and GxP Compliance: In a routine QC environment, 21 CFR Part 11 and Annex 11 compliance is non-negotiable. While lower-resolution systems are deeply integrated into standard chromatography data systems (CDS), modern QC-ready HRMS software packages now bring automated data handling and secure audit trails directly into standard CDS environments. Crucially, switching hardware and software platforms between analytical development (e.g., Orbitrap with Chromeleon) and commercial QC (e.g., single-quadrupole with alternative CDS) disrupts the digital thread and forces redundant computer system validations [13]. Retaining a unified HRMS ecosystem throughout the lifecycle is essential to re-evaluate historical full-scan data under ICH Q14 and ICH Q2(R2) principles.

Orbitrap platforms have been prevalent in the MAM due to their exceptional performance on mass accuracy, sensitivity, resolution, and mass stability, with modern benchtop units specifically designed for QC. TOF systems also offer good mass accuracy and resolution with high-speed acquisition needed to support rapid gradients or analyzing very large intact complexes. The use of TOF for the MAM has gained acceptance over the years [23,33]. Single quadrupole systems are more affordable and easier to maintain. However, its limited mass range prevents it from monitoring the full charge envelope of large glycopeptides. In addition, it lacks the NPD function necessary for detecting unexpected impurities, which poses a regulatory risk in replacing profile-based conventional QC methods. While lower-resolution MS platforms offer an attractive, budget-friendly operational footprint for simple targeted monitoring, the biopharma industry heavily favors HRMS for comprehensive and robust MAM deployment. This preference is driven by the necessity of high mass accuracy for reliable NPD and the increasing availability of QC-friendly, automated HRMS systems.

Ultimately, the choice of instrument depends on the intended purpose of the method, the cost-effectiveness, and the ease of use. At Roche/Genentech, a high-resolution instrument was selected based on the strategic requirement of the MAM, ultimately replacing multiple conventional methods.

### 3.2. The Use of MS/MS Capability in QC

In routine QC, many labs opt for MS1-only acquisition in QC for various reasons, including lower cost for a less complex instrument, simplified data processing, and potentially more robust, less variation between different tests due to more data points collected across narrow UHPLC peaks [12,13,30]. This MS1-only approach may particularly be beneficial for drug product (DP) applications where stability samples must be measured at different time points throughout a study across long intervals.

However, one of the most critical roles of MS/MS is as an investigative tool when the NPD algorithm flags an anomaly. If a new peak is detected, MS/MS fragmentation is used to determine if the peak is a true product-related degradant (e.g., a new oxidation site) or a sample preparation artifact (e.g., incomplete digestion or a plasticizer from a tube). Built-in MS/MS capabilities allow for immediate root-cause analysis. In instances where routine QC testing remains MS1-only without a built-in MS/MS capability, one would have to transfer the “failed” sample back to a characterization lab for identification, potentially delaying batch release.

While MS-1 only might be a currently more practical approach, MS/MS data acquisition and processing can be built in as part of the routine, automated, MAM data analysis in the future. This additional dimension of information should allow fast and confident investigation of unexpected impurities to support fast, real-time process monitoring and product release in the future.

### 3.3. MAM Beyond Peptide Map

While Peptide-level MAM remains the gold standard for detailed site-specific characterization, the intact and subunit-level MAM have emerged as compelling alternative solutions for high-throughput QC and at-line process monitoring. The strategic shift toward intact and subunit analysis is primarily driven by the need to reduce sample preparation-induced artifacts and significantly increase the speed of the “sample-to-answer” cycle.

The intact protein MAM (iMAM) involves the direct analysis of the whole, non-digested protein, providing a “bird’s-eye view” of global heterogeneity. It allows for rapid glycoform distribution assessment and total mass confirmation with virtually zero sample preparation, which minimizes the risk of introducing chemical modifications during processing. Complementing this, the subunit-level MAM utilizes specific enzymes like IdeS to cleave antibodies into smaller, manageable fragments (e.g., Fc/2, LC, and Fd’ fragments, about 25 kDa each). This “middle-down” approach offers a valuable compromise: it provides higher resolution than intact analysis—enabling the quantification of oxidation and C-terminal lysine clipping—without the complexity and artificial modifications, such as induced deamidation, often associated with the intensive digestion required for peptide mapping [13,26,33,45,46].

The utility of these higher-level approaches is also demonstrated in specialized applications. Kumar et al. reported the unique role of the intact/subunit MAM in monitoring the drug-to-antibody ratio (DAR) and conjugation site occupancy in antibody–drug conjugates (ADCs). These attributes are often difficult to quantify reliably at the peptide level due to the inherent hydrophobicity of the payloads [47]. In addition, complex MAM workflows have been established for the detailed structural characterization of subunit protein vaccines, monitoring critical parameters such as intact mass, sequence identity, protein clipping, glycosylation, other post-translational modifications, and host cell proteins (HCP) [48].

While the peptide-level MAM provides deep “site-specific” detail, iMAM for routine monitoring offers benefit because of its speed, reduced risk of sample preparation artifacts, and suitability for real-time integration.

### 3.4. MAM for Future Testing and Autonomous Manufacturing

At present, the MAM is primarily utilized as an off-line or at-line analytical tool for batch release, stability testing, and retrospective process characterization [13]. However, because the MAM allows for the direct, molecular-level evaluation of product quality, it provides a high-fidelity data source that is foundational for advanced quality by design (QbD) frameworks. The future vision for the MAM lies in its transition from an off-line assay into a fully integrated process analytical technology (PAT) to enable real-time release testing (RTRT). In this future state, the MAM would serve as a standardized “digital sensor” across global manufacturing networks, continuously feeding real-time CQA data into virtual process models, known as “digital twins” [49]. The bi-directional communication between the physical high-resolution mass spectrometer and the digital twin could allow manufacturers to predict outcomes and adjust bioreactor parameters autonomously. This convergence forms the conceptual basis for future autonomous manufacturing (the “Dark Factory”), where inspection and process adjustments are handled entirely by artificial intelligence (AI) agents without human intervention.

Despite this transformative potential, realizing the transition from current off-line or at-line monitoring to future autonomous RTRT faces several critical implementation challenges that must be addressed:

- Instrument and Software: Traditional QC laboratories are built around simpler assays. Validating robust LC-MS systems directly on the manufacturing floor requires immense effort to meet compliance guidelines. The MAM generates massive, complex datasets. Parsing this data in real-time requires advanced, validated, and 21 CFR Part 11-compliant software.
- Sample Preparation: Interfacing MS directly with upstream or downstream unit operations requires automated, robust, and extremely rapid online sampling. In-process materials, such as cell culture fluids, contain complex matrices that must be purified and digested in real-time without introducing artificial modifications (e.g., oxidation or deamidation), which is still a significant challenge.
- Data Processing: For autonomous control loops to function, data analysis must evolve from semi-automated, batch-processed workflows into fully autonomous, AI-driven systems. These systems must be capable of instantaneously deconvoluting spectra, quantifying CQA, and detecting new peaks.
- Model Validation and Life Cycle Management: While the ICH Q14 guideline explicitly supports RTRT, utilizing MS as a real-time release tool requires the complex validation of continuous predictive models. Maintaining these models, updating them for new batches, and establishing universal “model life cycle control practices” remain significant hurdles.

## 4. Conclusions

The implementation of multi-attribute method (MAM) represents a transformative paradigm shift in biopharmaceutical quality control, transitioning the industry from conventional profile-based assays to a comprehensive control strategy that utilizes TAQ and NPD. Successfully deploying this high-resolution LC-MS technology within a cGMP environment requires navigating significant technical and regulatory complexities. Current industry best practices for MAM implementation rest on four foundational pillars: establishing compliance-ready informatics and rigorous instrument qualification to ensure data integrity, shifting to proactive and strictly scheduled instrument maintenance, developing comprehensive SST using product-specific reference standards, and adopting phase-appropriate validation strategies. Furthermore, the successful global transfer of these complex methods relies heavily on standardizing centralized informatics and utilizing robotic liquid handling systems to mitigate analyst and instrumental variability.

Ultimately, the potential impact of the MAM extends far beyond streamlining routine QC workflows and reducing laboratory footprints. By providing granular, residue-level data on product quality attributes, the MAM serves as a physical realization of the QbD framework, moving the industry from a reactive “testing-to-document” to a proactive “testing-to-control” approach. As the MAM continues to evolve—incorporating rapid intact and subunit-level analyses—it is poised to act as a critical “digital sensor” in next-generation manufacturing. By seamlessly integrating real-time molecular data with virtual “digital twins,” the MAM will directly facilitate real-time release testing (RTRT) and autonomous manufacturing process controls, accelerating the delivery of complex, life-saving therapeutics to patients.

## Figures and Tables

**Table 1:** Commercially available MAM software platforms.

Vendor	Software Solution(s)	Compliance Readiness
Thermo Scientific	Chromeleon CDS & BioPharma Finder	Enterprise GxP ^1^
Waters	Waters connect (Peptide MAM App)	Full GxP ^2^
Sciex	SCIEX OS & Biologics Explorer	Full GxP ^2^
Genedata	Genedata Expressionist	Enterprise GxP ^1^

^1^ enterprise GxP is the highest level of readiness. It uses a centralized database (e.g., Oracle or SQL Server) so data is not stored locally on the mass spectrometer computer. It features global user management, centralized backup/archiving, and high availability. ^2^ full GxP software meets 21 CFR Part 11 and Annex 11 requirements. It includes comprehensive audit trails, electronic signatures, version control, and “Reason for Change” prompts. The scope is typically a single workstation or a specific laboratory site.

**Table 2:** System suitability testing parameters and performance demonstration for TAQ and NPD.

Parameter	Performance Characteristics
Mass accuracy of 4 selected peptides	Mass accuracy
Signal intensity for 3 selected peptides eluted across run time	MS instrument sensitivity Recovery from LC Recovery from sample preparation LC retention time
Relative abundance of an alkylated peptide	Alkylation MS relative quantitation
Relative abundance of a low-level, oxidized peptide	MS instrument sensitivity MS relative quantitation Stability of oxidized peptides Column aging
Visual inspection of profile	Entire workflow performance
BPC ratio of the bracket reference standard injections and for each sample injection	Signal stability for NPD
NPD between bracketing reference standard	Specificity for NPD

**Table 3:** Performance characteristics and acceptance criteria (for impurities).

Performance Characteristics	Acceptance Criteria
**TAQ Characteristics**	
Specificity	Interference ≤ 1%
Response (Linearity)	r ≥ 0.98
Accuracy by recovery	80–120%
Repeatability	RSD ≤ 15%
Intermediate precision	RSD ≤ 20%
QL	Attribute-specific, S/N ≥ 10
Range	Attribute-specific
Stability of prepared sample (change in abundance) at 24 and 48 h	Evaluation of change in abundance at 24 and 48 h
**NPD Characteristics**	
Specificity	No new peak between reference standard injections. Expected new peak and no other new peak detected in model sample
Detection Limit	Expected new peak detected in model sample
Intra-lab Comparability	Expected new peak and no other new peak detected in model sample
Stability of prepared sample at 24 and 48 h	No new peak between reference standard injections. Expected new peak and no other new peak detected in model sample

## Data Availability

No new data were created or analyzed in this study. Data sharing is not applicable to this article.
